# The grapevine kinome: annotation, classification and expression patterns in developmental processes and stress responses

**DOI:** 10.1038/s41438-018-0027-0

**Published:** 2018-04-01

**Authors:** Kaikai Zhu, Xiaolong Wang, Jinyi Liu, Jun Tang, Qunkang Cheng, Jin-Gui Chen, Zong-Ming (Max) Cheng

**Affiliations:** 1 College of Horticulture, Nanjing Agricultural University, Nanjing, Jiangsu 210095 China; 2 Department of Plant Sciences, University of Tennessee, Knoxville, TN 37996 USA; 3 Jiangsu Key Laboratory for Horticultural Crop Genetic Improvement, Institute of Horticulture, Jiangsu Academy of Agricultural Sciences, Nanjing, Jiangsu 210014 China; 4 Department of Entomology and Plant Pathology, University of Tennessee, Knoxville, TN 37996 USA; 5 Biosciences Division, Oak Ridge National Laboratory, Oak Ridge, TN 37831 USA

## Abstract

Protein kinases (PKs) have evolved as the largest family of molecular switches that regulate protein activities associated with almost all essential cellular functions. Only a fraction of plant PKs, however, have been functionally characterized even in model plant species. In the present study, the entire grapevine kinome was identified and annotated using the most recent version of the grapevine genome. A total of 1168 PK-encoding genes were identified and classified into 20 groups and 121 families, with the RLK-Pelle group being the largest, with 872 members. The 1168 kinase genes were unevenly distributed over all 19 chromosomes, and both tandem and segmental duplications contributed to the expansion of the grapevine kinome, especially of the RLK-Pelle group. *Ka/Ks* values indicated that most of the tandem and segmental duplication events were under purifying selection. The grapevine kinome families exhibited different expression patterns during plant development and in response to various stress treatments, with many being coexpressed. The comprehensive annotation of grapevine kinase genes, their patterns of expression and coexpression, and the related information facilitate a more complete understanding of the roles of various grapevine kinases in growth and development, responses to abiotic stress, and evolutionary history.

## Genetics: Mapping molecular switches in grape

A comprehensive map of all the protein kinases (PKs) in grape is now available. PKs switch other proteins on or off by adding or removing a phosphate group. They regulate many cellular processes, coordinate plant growth, and trigger responses to various stresses. However, the number, relationships, and functions of PKs remain uncharacterized in most plants. Zong-Ming Cheng at Nanjing Agricultural University and the University of Tennessee mapped all the PKs in grape. Using the most up-to-date version of the genome, Cheng and coworkers identified over 1000 PKs, traced their evolutionary relationships, and identified which PKs are active in key growth phases. They also identified many PKs involved in response to stresses such as heat and drought. These results may help to improve stress tolerance in one of the world’s most economically important fruits.

## Introduction

Reversible phosphorylation, catalyzed by protein kinases (PKs), is a type of post-translational modification that exists widely in both prokaryotic and eukaryotic organisms^1^. PKs constitute one of the largest gene families in eukaryotic genomes and are involved in numerous cellular and biological processes^2,3^. The majority of PKs transfer the γ-phosphate from adenosine triphosphate (ATP) to phosphorylate specific amino acids, mainly serine (S), threonine (T), or tyrosine (Y), in order to regulate the activity of their target proteins^4^. Individual PK genes have been extensively analyzed in many species, including both prokaryotic and eukaryotic organisms and ranging from animals to plants in the latter group^5^. The first plant protein kinase was isolated from pea shoots (*Pisum sativum*) in 1973^6^, and the first plant PK DNA sequences were identified in bean (*Phaseolus vulgaris*) and rice (*Oryza sativa*) by using degenerate primers^7^. Since then, many plant PKs and PK gene families have been identified in plants, including *Arabidopsis*^5,8,9^, maize^10^, soybean^3,11^, tobacco^12^, and apple^13^.

The kinome, defined as the entire collection of kinase genes in a genome, was reported in *Arabidopsis* in 2004 as containing over 1000 PKs^14^ and in rice as including ~1500 protein kinases^15^. Rice kinome meta-expression data provided evidence that rice PKs have functional roles in development and in stress tolerance^16^. More than 2000 kinase genes make up the soybean kinome, which is approximately twice as many as that in *Arabidopsis* and four times the size of the human kinome^3^. In general, however, the number of protein kinases in plants is much greater than those in humans and animals^17,18^. The expansion of protein kinases in plants may be the result of recent duplication events, whole-genome duplication, segmental duplication, tandem duplication, or all three^19^.

Protein kinases generally possess a catalytic domain, which is composed of 250-300 amino acid residues^2^. Classification of PKs was initially based on a phylogenetic analysis of the kinase domain^20^. Lehti-Shiu and Shiu^2^ classified the PKs of 25 sequenced plant species into 9 major groups including AGC (PKA-PKG-PKC), CAMK (calcium- and calmodulin-regulated kinase), CK1 (casein kinase 1), CMGC (cyclin-dependent kinases, mitogen-activated protein kinases, glycogen synthase kinases and cyclin-dependent like kinases), STE (sterility), TK (tyrosine kinase), TKL (tyrosine kinase-like kinase), and plant-specific.

Grape (*Vitis vinifera* L.) is one of the most popular and economically important fruits in the world^21,22^. Grape production, similar to that of other crops, however, must address abiotic and biotic stresses, which cause reductions in yield and fruit quality. The publication of the grapevine genome^23^ has provided tremendous new opportunities for comprehensive transcriptomic and proteomic studies, as well as other genetic and molecular studies. Information on several grapevine kinase gene families has been reported, including mitogen-activated protein kinase (MAPK)^24^, Ca^2+^-dependent protein kinase (CDPK)^25^, and sucrose non-fermenting 1-related protein kinase 2 (SnRK2)^26^. In addition, a preliminary analysis of the entire superfamily of grapevine protein kinases was first reported in 2012; 877 grapevine kinase genes were identified and classified into 118 families^2^. In the current study, 1168 putative PK coding genes were identified in the most recent grapevine genome database and classified into groups and families. We further analyzed the distribution of kinases in the grapevine genome, intron numbers, subcellular location prediction, expansion mechanisms, and gene expression patterns in different tissues during development and in response to a variety of stress treatments. In addition, a coexpression network was constructed. This study provides a comprehensive overview of the entire grapevine kinome and the potential roles of different groups of kinases in grapevine growth and stress response and adaptation.

## Materials and methods

### Computational identification and classification of grapevine protein kinases

Grapevine (*Vitis vinifera*) predicted proteins (Version 2.1) were downloaded from the Grape Genome Biotechnology Center (CRIBI, http://genomes.cribi.unipd.it) and used to identify the entire kinome of grapevine. Files containing Hidden Markov Models (HMMs) of the Pkinase clan (Pkinase (PF00069) and Pkinase_Tyr (PF07714)) were downloaded from Pfam (http://pfam.xfam.org/) and used to identify putative protein kinases^27^ using HMMER v. 3.1b1 software with an E-value cutoff of <1.0^28^. The presence of a kinase domain in each of the candidate PK genes was verified using Pfam and SMART (http://smart.embl-heidelberg.de/). Subsequently, each sequence containing the protein kinase domain was extracted, and redundant sequences were deleted. Only the longest peptide sequence of each gene was used in cases where an identified gene contained more than one predicted isoform. This approach resulted in the identification of a total of 1358 grapevine protein kinases. An identified sequence was considered a putative protein kinase if the kinase domain alignment covered at least 50% of the Pfam domain model^2,3^. This selection criterion resulted in the identification of a final total of 1168 proteins containing at least one kinase domain (Supplementary Table S1) and identified as grapevine protein kinases. The identified grapevine PKs were then classified into different groups and families using HMMs of the different kinase families developed from the PK sequences of 25 plant species^2^.

### Multiple sequence alignment and phylogenetic analysis

The kinase domain sequences of all 1168 grapevine PKs were aligned using MUSCLE, and a phylogenetic tree was constructed using the Neighbor-Joining (NJ) method in MEGA 6.06^29^. The parameter pairwise deletion and the Poisson model were used with 1000 bootstrap replicates.

### Intron numbers and chromosomal locations

The chromosomal positions of grapevine PK genes were collected from CRIBI. Intron numbers of all of the grapevine kinase genes were obtained from the GFF file from CRIBI.

### Protein properties and subcellular localization prediction

The molecular weight (MW) and isoelectric point (pI) for all of the identified grapevine PK protein sequences were predicted using the ExPASy server tool (http://web.expasy.org/compute_pi/)^30^. The predicted subcellular localizations of the grapevine PKs were determined using the online tool WoLF PSORT (http://www.genscript.com/wolf-psort.html)^31^.

### Identification of tandem and segmental duplication events

Tandem duplications of grapevine kinase genes were predicted by determining their physical location on grapevine chromosomes. An array of two or more genes located on the same chromosome and separated by five or fewer genes in a 100-kb region was considered to represent a tandem duplication event^32^. An illustration of the chromosomal locations of tandemly duplicated genes was constructed using Mapchart 2.30 (http://www.wur.nl/en/show/Mapchart-2.30.htm). The segmental duplication events of grapevine kinases were determined using the MCScanX (Multiple Collinearity Scan, http://chibba.pgml.uga.edu/mcscan2/) package^33^.

### GO analysis of tandemly duplicated *PK* genes

The Gene Ontology (GO) term ID of each tandemly duplicated PK gene in grapevine was retrieved from the CRIBI database (http://genomes.cribi.unipd.it/DATA/V2/annotation/bl2go.annot.txt). The annotations of the GO term IDs were collected from the Gene Ontology Consortium (http://www.geneontology.org).

### Calculations of synonymous substitutions (*Ks*) and non-synonymous substitutions (*Ka*) and selection modes

The coding sequences (CDS) of the tandemly and segmentally duplicated grapevine *PK* genes in each family were aligned using ClustalW 2.0^34^ in order to detect the selection modes of the tandem and segmental duplication events. The *Ka*, *Ks*, and ratio of *Ka* to *Ks* (*Ka*/*Ks*) were calculated using MEGA 6.06^29^. The approximate divergence times (T) of the duplication events were calculated using the equation T = *Ks*/2*λ* × 10^-6^ MYA (million years ago), in which the mean *Ks* rate *λ* = 6.5 × 10^−9^ for grapevine^35^.

### Microarray data analysis

Genome-wide expression data for grapevine from the Gene Expression Omnibus (GEO) database (https://www.ncbi.nlm.nih.gov/geo/) at the NCBI website (https://www.ncbi.nlm.nih.gov/) were analyzed for the expression patterns of grapevine *PK* gene families in different tissues and developmental stages and in response to different treatments. The GSE36128 dataset provided gene expression profiling of 54 diverse grapevine samples covering different growth stages of most grapevine organs^21^. The expression data of 1,109 available grapevine kinase genes were collected and normalized. The gene expression data of 231 available kinase genes in response to salt, polyethylene glycol (PEG), cold, drought, heat and abscisic acid (ABA) treatments were downloaded from four selected Affymetrix datasets, GSE31594, GSE31677, GSE31675, and GSE31662. These datasets were used to analyze the expression of grapevine PKs in response to different stress treatments. The expression data on the response of *Arabidopsis* PK genes to drought (GSE65414) and salt (GSE71855) were also retrieved to compare gene expression between the grapevine kinome and the *Arabidopsis* kinome. Expression data were analyzed using the GEO2R online tool (https://www.ncbi.nlm.nih.gov/geo/geo2r/). To analyze the expression data at the family level, the normalized gene expression data of all members in each family were averaged. Since expression data for only 1109 of the 1168 identified grapevine kinase genes was available in 54 different tissues and the responses of only 231 of the 1168 grapevine PKs to different stress treatments were available, only families where at least 33% of the family gene members were represented in the expression data were considered^3^. Heatmaps representing the expression data of 120 families in different tissues at different developmental stages and the responses of 56 families to different stress treatments were constructed using the R package (https://www.r-project.org/) pheatmap.

### Coexpression network of grapevine PK families

A coexpression network of 56 grapevine PK families in response to six different treatments was constructed based on Pearson correlation coefficients (PCCs) to better understand the topological relationships between stress-responsive grapevine PK families. All of the available gene expression data in each family were averaged, and the PCC was calculated between each pair of families using the method described by Tang et al^36^. All of the grapevine PK family pairs with PCCs at 0.05 significance level (*p*-value) were extracted and used to illustrate a coexpression network using Cytoscape 3.3 (http://www.cytoscape.org).

### Plant materials, growth conditions and treatments

In vitro-grown grapevine plants (*Vitis vinifera*, Pinot noir 40024, the sequenced genotype) were maintained on half-strength Murashige and Skoog medium containing 0.3 mg/L indole 3-butyric acid (IBA) and grown at 25 °C in the culture room under a photoperiod of 16/8 h and a light intensity of 100 μmol m^−2^ s^−^^1^. Five-week-old tissue-cultured grapevine plants were transplanted into pots and acclimatized in a growth chamber with 16 h light at 24 °C/8 h dark at 22 °C and 70–80% relative humidity. Plants grown in pots were well watered for the first four weeks, and then, water was withheld to impose drought treatment. Grapevine leaf samples were collected at 0 (control), 2, 4, and 8 days after water was withheld. For salt treatment, in vitro-grown 5-week-old plants were irrigated with 200 mM NaCl directly and grown at 25 °C under a photoperiod of 16/8 h. Leaves were collected at 0 (control), 6, 12, and 24 h and used in real-time quantitative reverse transcription PCR (RT–PCR) analysis. All the collected samples were immediately frozen in liquid nitrogen and stored at −70 °C for further analysis. Three biological replicates were used for each treatment or control.

### RNA isolation and real-time quantitative RT–PCR analysis

Total RNA was extracted from grapevine leaves using the Plant Total RNA Isolation Kit (Foregene, Chengdu, China) according to the manufacturer’s instructions. The genomic DNA was removed from the sample using DNase I (RNase-free DNase set, Qiagen, Hilden, Germany), and RNA concentration and quality were measured by determining the optical density (OD) ratios at 260 nm and 280 nm with a One Drop OD-1000 spectrophotometer (Thermo Scientific, Wilmington, DE). Then, first-strand cDNA was synthesized from 1 μg total RNA using Prime Script RT Reagent Kit (Takara, Japan) according to the manufacturer’s instructions. Specific primers for the grapevine PK genes were designed using the IDT PrimerQuest tool (https://www.idtdna.com/PrimerQuest/Home/Index). The actin gene (*VIT_212s0178g00200*) was used as a reference gene for data normalization; we have confirmed this to be an appropriate reference gene^24,26^. The qRT–PCR was conducted using a LightCycler 480 SYBR Green I Master (Roche, Mannheim, Germany) on a Roche 480 Real-Time PCR System (Roche, Mannheim, Germany). The qRT–PCR was performed according to the manufacturer’s protocol, and the total volume of each reaction was 15 μl. The PCR conditions used were as follows: 95 °C 10 min, 40 cycles of 10 s at 95 °C, 20 s at 58 °C, and 10 s at 72 °C. At the end, a melting curve was generated from 65 to 97 °C. The relative gene expression levels in stressed plants relative to control were shown using the ∆∆CT method^11^. Values were means ± SE calculated from the three biological replicates.

### RNA-Seq analyses of grapevine PK genes

Three biological replicates of grapevine leaf samples under the aforementioned drought treatment were collected and used for an RNA-Seq experiment. A total amount of 1 μg RNA per sample was used as input material for the RNA library preparations. Sequencing libraries were generated using NEBNext UltraTM RNA Library Prep Kit for Illumina (NEB, USA) following manufacturer’s recommendations. The transcript abundance of the grapevine kinase genes was calculated as fragments per kilobase of exon model per million mapped reads (FPKM). The RNA-Seq raw data have been uploaded to the Sequence Read Archive of NCBI (https://www.ncbi.nlm.nih.gov/sra/) with accession number PRJNA433817.

### Statistical analysis

Statistical analysis was performed using either *t*-test (*P < *0.05) or Duncan’s multiple range test (*P* < 0.05) where appropriate using IBM SPSS Statistics v24 software (SPSS, Inc., USA).

## Results

### Genome-wide identification and classification of grapevine protein kinases

**Fig. 1:**
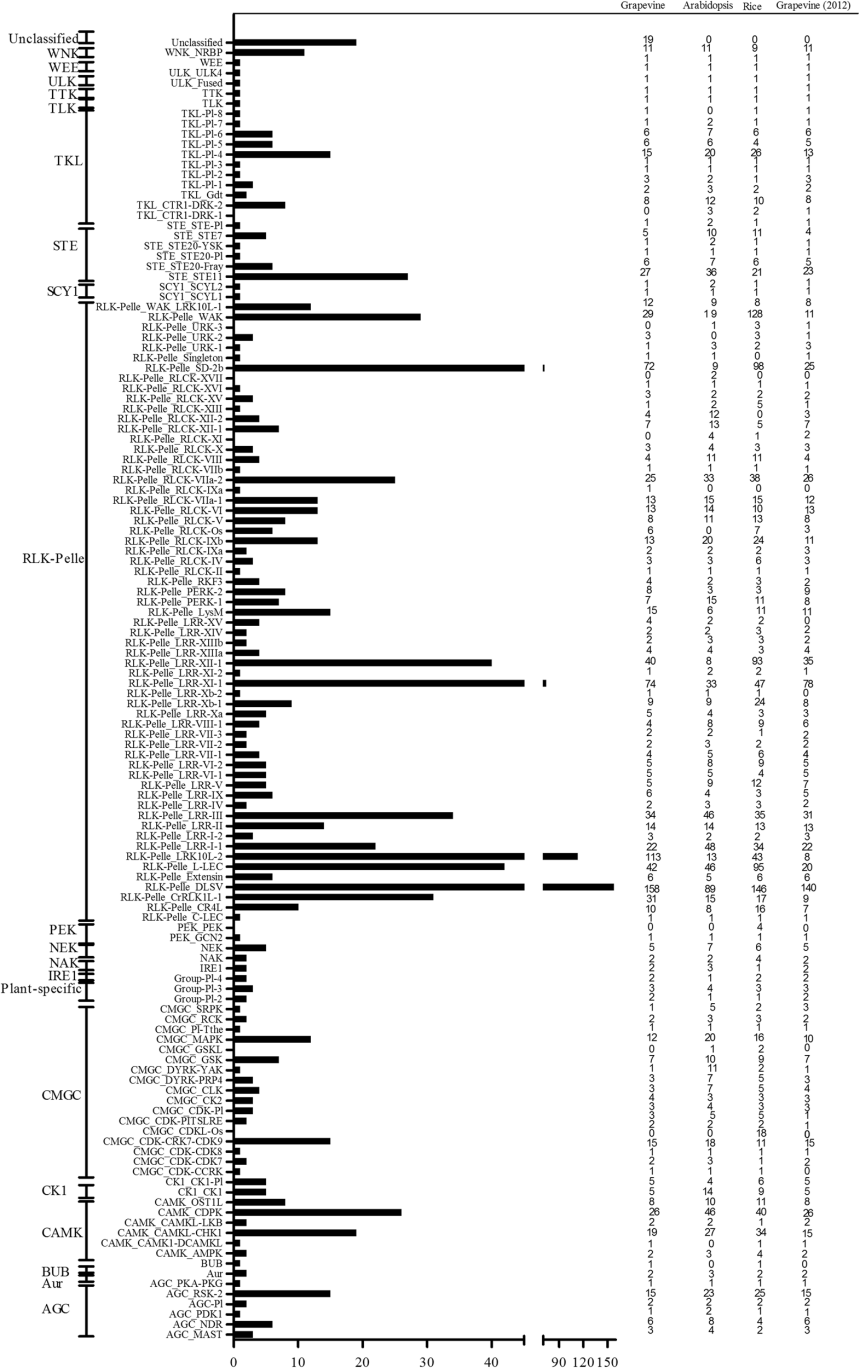
Comparison of grapevine protein kinase families with those in *Arabidopsis* (eudicot species) and rice (monocot species). The grapevine PKs were classified into 20 groups and 121 families

The putative PK sequences in the grapevine genome database were identified by HMMER and aligned to the Pfam kinase domain. A total of 1,168 typical putative grapevine PK genes (kinase domain aligned with at least 50% of the kinase domain models; Supplementary Table S1) and 190 atypical PKs (Supplementary Table S2) were identified. The 1,168 typical PKs were classified (Fig. 1; Supplementary Table S3) and further examined using a phylogenetic analysis (Supplementary Fig. S1; Supplementary Fig. S2). The results of the phylogenetic analysis indicated that only 19 of the 1168 genes exhibited a classification that was different from the HMM search result. These 19 genes had low E-values and did not cluster with any other known families. Thus, they were placed in an unclassified cluster. The remaining 1149 grapevine PKs were classified into 20 groups, which included AGC, Aur, BUB, CAMK, CK1, CMGC, IRE1, NAK, NEK, PEK_GCN2, RLK-Pelle, SCY1, STE, TKL, TLK, TTK, ULK, WEE, WNK, and plant-specific (Fig. 1). Some of the larger groups were further classified into different families within each group^2,3^. The overall classification resulted in 20 groups and 121 families (Fig. 1; Supplementary Table S3). The RLK-Pelle group was the largest group, accounting for 74.66% (872/1168) of the grapevine *PK* genes.

In comparison to the classifications of *Arabidopsis* and rice and to the grapevine kinomes reported previously^2^, 35 grapevine families contained only 1 member, including AGC_PKA-PKG, CMGC_CDK-CCRK, CMGC_CDK-CDK8, CMGC_Pl-The, RLK-Pelle_RLCK-II, and TTK (Fig. 1). Most of these grapevine PKs were also conserved in the rice and *Arabidopsis* kinomes. Surprisingly, the RLK-Pelle_LRK10L-2 and RLK-Pelle_LRR-XI-1 families contained many more genes in grapevine than in *Arabidopsis* and rice, indicating that these two families have expanded greatly in grapevine. However, the RLK-Pelle_LRK10L-2 family (8) was much smaller^2^. Five families were newly found in the grapevine kinome, including BUB, CMGC_CDK-CCRK, RLK-Pelle_LRR-Xb-2, RLK-Pelle_LRR-XV, and RLK-Pelle_RLCK-IXa (Fig. 1). The monocot-specific kinase families (GMGC_CDKL-Os, 18 members) were not found in either *Arabidopsis* or grapevine^3^. RLK-Pelle_RLCK-IXa contained only one member in grapevine and was absent in rice and *Arabidopsis*.

### The kinase domains, intron numbers, protein properties, and predicted subcellular localizations of grapevine PKs

The sequence of the kinase domain, intron number, and protein properties, including molecular weight (MW) and isoelectric point (pI) (Supplementary Table S3), were characterized for each of the 1168 grapevine PKs. Most (95.21%) grapevine protein kinases possess only one catalytic domain, 54 possess two catalytic domains, and 2 possess three kinase domains (VIT_209s0002g02840 and VIT_209s0002g02840) (Supplementary Table S4). The 56 grapevine protein kinases with two or more kinase domains were distributed in 18 different families. The average intron number in the 1,168 kinase genes was 5, with 194 PK genes being intronless genes, of which 173 were RLK-Pelle genes. The number of introns per gene varied from 0 to 49 (*VIT_212s0055g00590*). Only 147 (12.59%) of the grapevine kinase genes contained more than 10 introns, and 26 (2.23%) contained more than 20 introns. The pIs of the grapevine PKs varied from 4.48 to 10.19, with MWs ranging from 16,243.53 to 230,303.18 daltons (Supplementary Table S3). In general, grapevine PK genes within the same families shared similar numbers of introns, as well as similar pIs and MWs.

The subcellular localizations of the grapevine PKs were determined using WoLF PSORT software (Supplementary Table S3). Most of the PKs (297) were predicted to localize to the plasma membrane, whereas 223, 225, and 248 were predicted to reside in the cytoplasm, nucleus, and chloroplast, respectively. Several families were also predicted to have the same subcellular locations for all members. For example, AGC_MAST, CK1_CK1, CMGC_CDK-PITSLRE, and TKL-Pl-6 family members were all predicted to localize to the nucleus. Members of the Aur, CAMK_AMPK, CMGC_CDK-Pl, and Group-Pl-4 families were all predicted to be cytoplasmic. Many other PKs within the same families, however, were predicted to localize to different cellular compartments.

### Chromosomal locations and tandem duplications of grapevine *PK* genes

Almost all of the PK genes (1063/1168) could be mapped to the 19 grapevine chromosomes (Supplementary Table S3), but they are not evenly distributed among the 19 chromosomes. Chromosome 16 hosts the largest number (107) of *PK* genes, whereas chromosome 2 has the fewest (23). Grapevine PK members in the same family are generally distributed on different chromosomes. Interestingly, most RLK-Pelle_LRK10L-2 gene family members are located on chromosome 16, and the RLK-Pelle_LRR-I-1 gene family members are mainly distributed on chromosome 9. All of the RLK-Pelle_RLCK-XII-2 gene family members are located on chromosome 18.

**Fig. 2:**
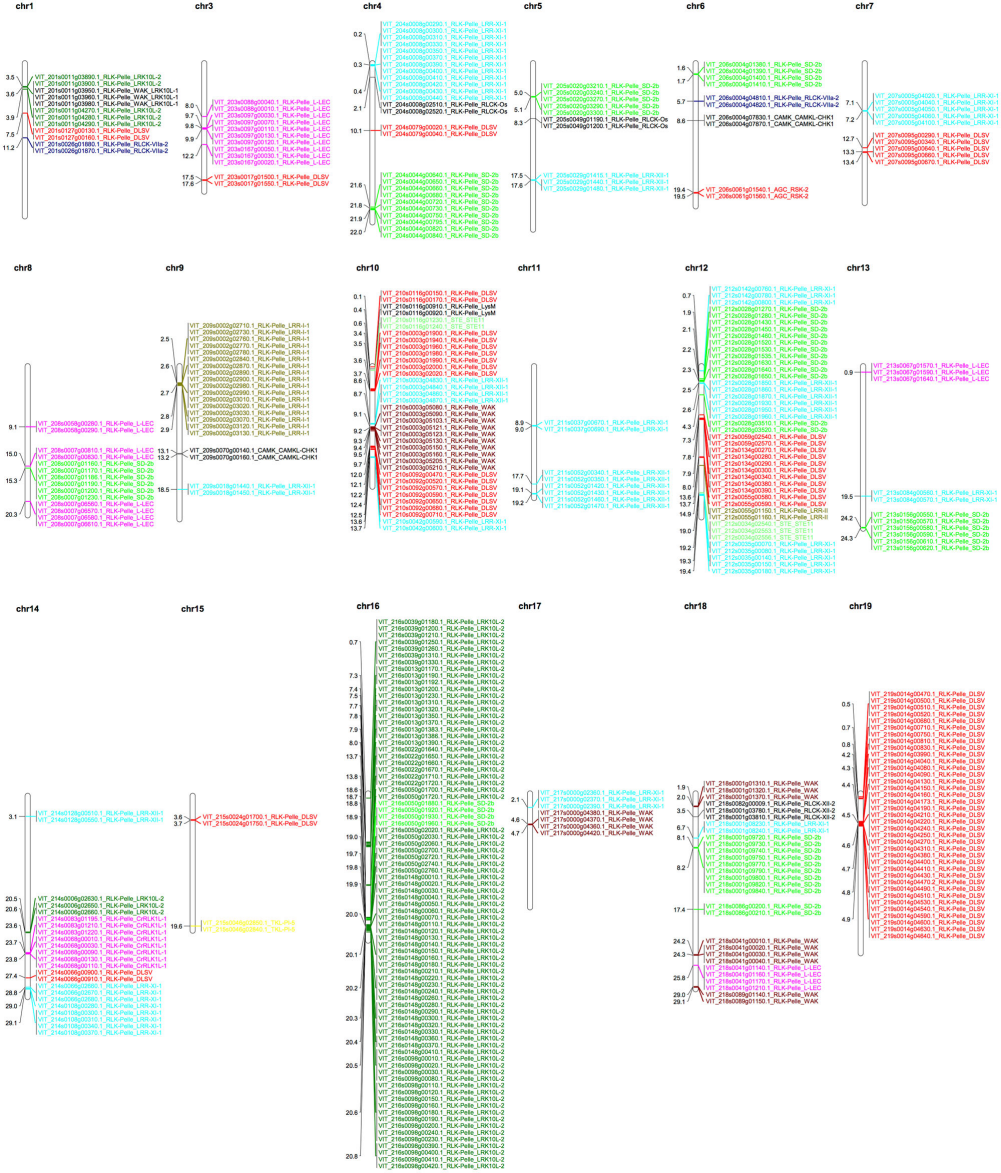
Chromosomal locations of tandemly duplicated kinase genes in grapevine. A total of 395 tandemly duplicated kinase genes representing 19 families were distributed unevenly among 18 chromosomes. Gene IDs and related family names are presented to the right of each chromosome, and related information on gene location is listed on the left

Grapevine PK genes are tandemly arranged on chromosomes based on their physical distribution. It appears that 395 PK genes were generated by tandem duplication (Fig. 2; Supplementary Table S5). These tandem duplication events occurred in 19 families that were unevenly distributed across all of the chromosomes with the exception of chromosome 2. Chromosome 16 possesses the greatest number (82) of PK genes, and 78 of these are RLK-Pelle_LRK10L-2 genes. Tandem duplication events were responsible for more than 60% of the expansion in four families, RLK-Pelle_LRK10L-2 (76.11%), RLK-Pelle_LRR-I-1 (77.27%), RLK-Pelle_RLCK-Os (66.67%), and RLK-Pelle_RLCK-XII-2 (75%), all of which belong to the RLK-Pelle group.

**Fig. 3:**
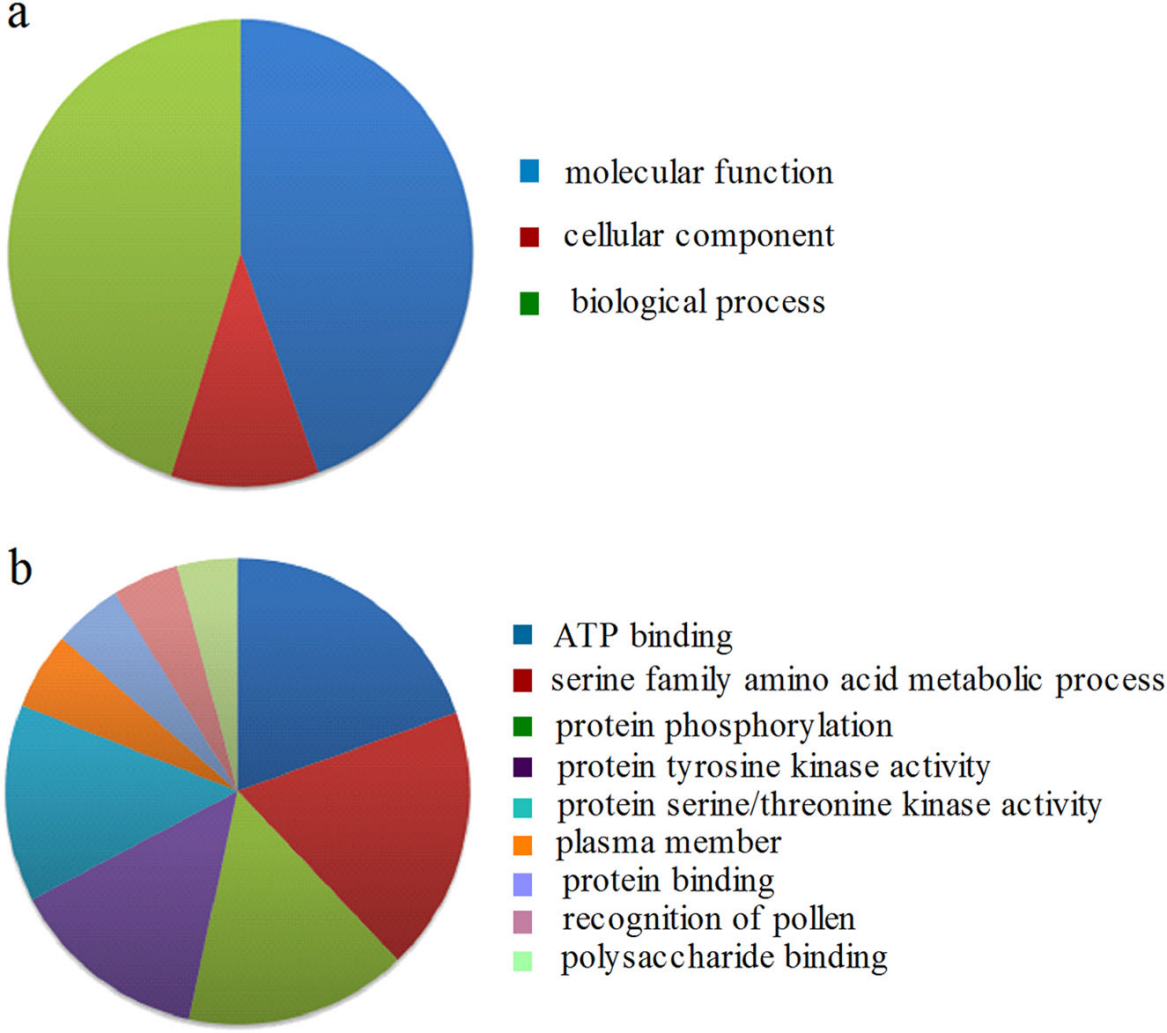
Gene Ontology (GO) analysis of tandemly duplicated grapevine *PK* genes. The size of each slice in the pie chart indicates the relative abundance of that GO term in the grapevine kinome. GO terms were collected from the CRIBI database

Previous studies have indicated that genes that are duplicated by tandem duplication events may play distinct roles in plant development and response to abiotic and biotic stresses^37^. Therefore, the GO annotations for 395 tandemly duplicated grapevine PK genes were examined (Fig. 3). The three main GO categories are biological process, cellular component, and molecular function. The majority of grapevine PKs that arose by tandem duplication were enriched in two of the three GO categories, namely, biological process and molecular function, with only approximately 10% of tandemly duplicated PK genes enriched in the GO category of cellular component (Fig. 3a). Functional GO terms for the 395 tandemly duplicated grapevine PK genes were also assessed. The top two terms were ATP binding (GO:0005524) and serine family amino acid metabolic process (GO:0009069; Fig. 3b). These results suggest that these PKs are associated with multiple biological processes, as well as signaling and response to stress.

### *Ka* and *Ks* calculations and selection modes of tandem and segmental duplication events

In addition to tandem duplication events, which were the main contributors to the expansion of grapevine PKs, segmental duplication events played a role. Collectively, tandem and segmental duplication events were identified (Supplementary Table S6). These segmental duplication events include 231 PK genes from 47 families, located on all 19 chromosomes.

To determine the selection types and divergence dates of the tandem and segmental duplication events associated with the grapevine PK genes, the synonymous (*Ks*) and non-synonymous substitutions (*Ka*) between the gene pairs were determined. The substitution ratio of *Ka* to *Ks* was calculated in order to evaluate the selection model of the gene pairs. *Ka/Ks < *1 indicates purifying selection on the gene pairs, *Ka/Ks* = 1 indicates neutral selection, and *Ka/Ks > *1 indicates that the gene pairs are under positive selection^38^. A summary of the *Ka/Ks* ratios for the 585 tandem and 138 segmental duplication events is shown in Supplementary Table S6. The *Ka/Ks* values in 101 of the segmentally duplicated gene pairs were less than 1. Only 37 of the gene pairs were under positive selection, and all of these were members of the RLK/Pelle group. Similar results were observed for the PK genes associated with tandem duplication events. In this case, the *Ka/Ks* values of 541 of the 585 gene pairs were less than 1. Collectively, these data indicate that the majority of tandemly and segmentally duplicated genes in the grapevine kinome were subjected to purifying selection (Supplementary Fig. S3; Supplementary Table S6). The divergence dates of the tandemly and segmentally duplicated gene pairs occurred from 0.30 to 387.18 and 0.35 to 374.66 MYA, respectively (Supplementary Table S6).

### Gene expression patterns of grapevine PKs in different tissues

**Fig. 4:**
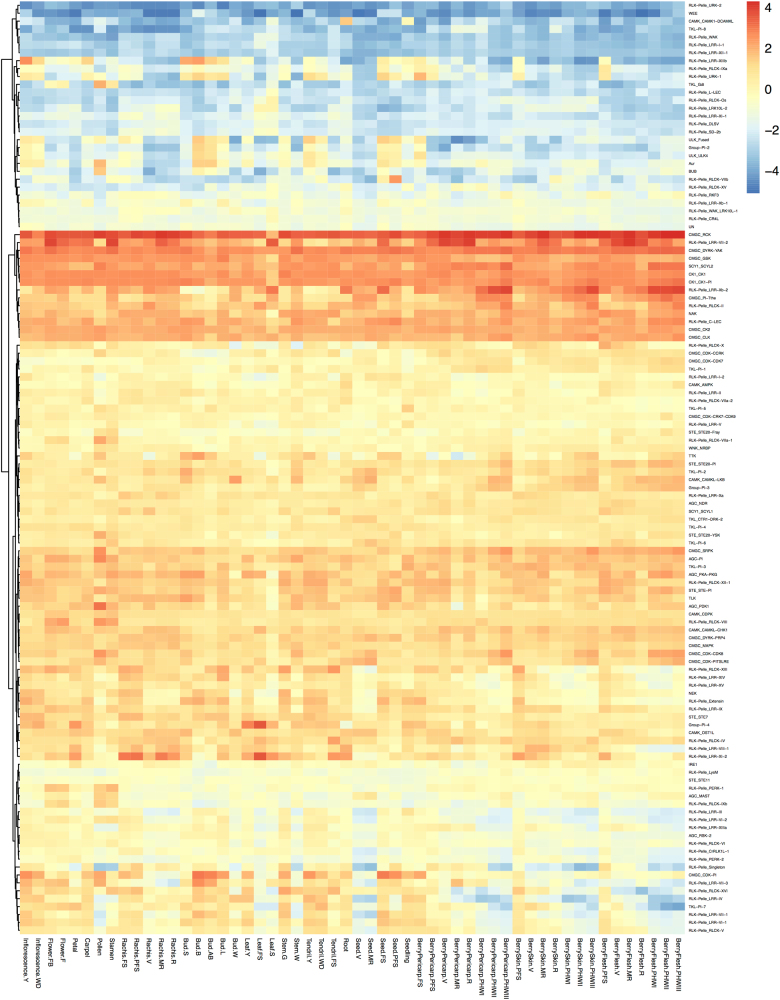
A heatmap illustrating the expression data of 120 different grapevine kinase families in 54 different grapevine tissues at various stages of development. The heatmap was generated using the R package pheatmap. The color scale represents expression levels, with red indicating high expression levels and blue indicating low levels

Protein kinases have been reported to play critical roles in plant development^39,40^. Therefore, the expression profiling data of 54 different grapevine tissues and organs during different developmental stages (accession #GSE36128) were analyzed to obtain insight into the temporal transcription patterns of grapevine PK genes during development. Expression data for 1,109 of the 1,168 grapevine PK genes were available, and the expression of these genes in the acquired datasets was analyzed at the PK family level (Supplementary Table S7). Cluster analysis of the microarray data reflecting the expression of 120 PK families was calculated in the 54 different tissues (Fig. 4). The results indicated that different families exhibit different expression patterns during plant development. Most families in the AGC, CAMK, CMGC, SCY1, STE, and TKL groups exhibited high expression levels in most tissues, suggesting that these families play key roles in developmental processes. In contrast, Aur, BUB, ULK, WEE and most families in the RLK-Pelle groups exhibited very low expression levels in most of the different tissues (Fig. 4). Although the RLK-Pelle group contains 58 families, only RLK-Pelle_C-LEC, RLK-Pelle_LRR-VII-2, RLK-Pelle_LRR-Xb-2, and RLK-Pelle_RLCK-II exhibited high expression levels in nearly all 54 tissues. Other families had high levels of expression in specific tissues. For example, RLK-Pelle_RLCK-VIIb exhibited high levels of expression in seed-PFS (post fruit set seed), and RLK-Pelle_PERK-1, RLK-Pelle_RLCK-IXb, RLK-Pelle_RLCK-VIIa-1, and RLK-Pelle_RLCK-VIII had high expression levels in flower, pollen, and stamens (Fig. 4).

### Microarray analyses and the related coexpression network of grapevine PK families in response to different stress treatments

**Fig. 5:**
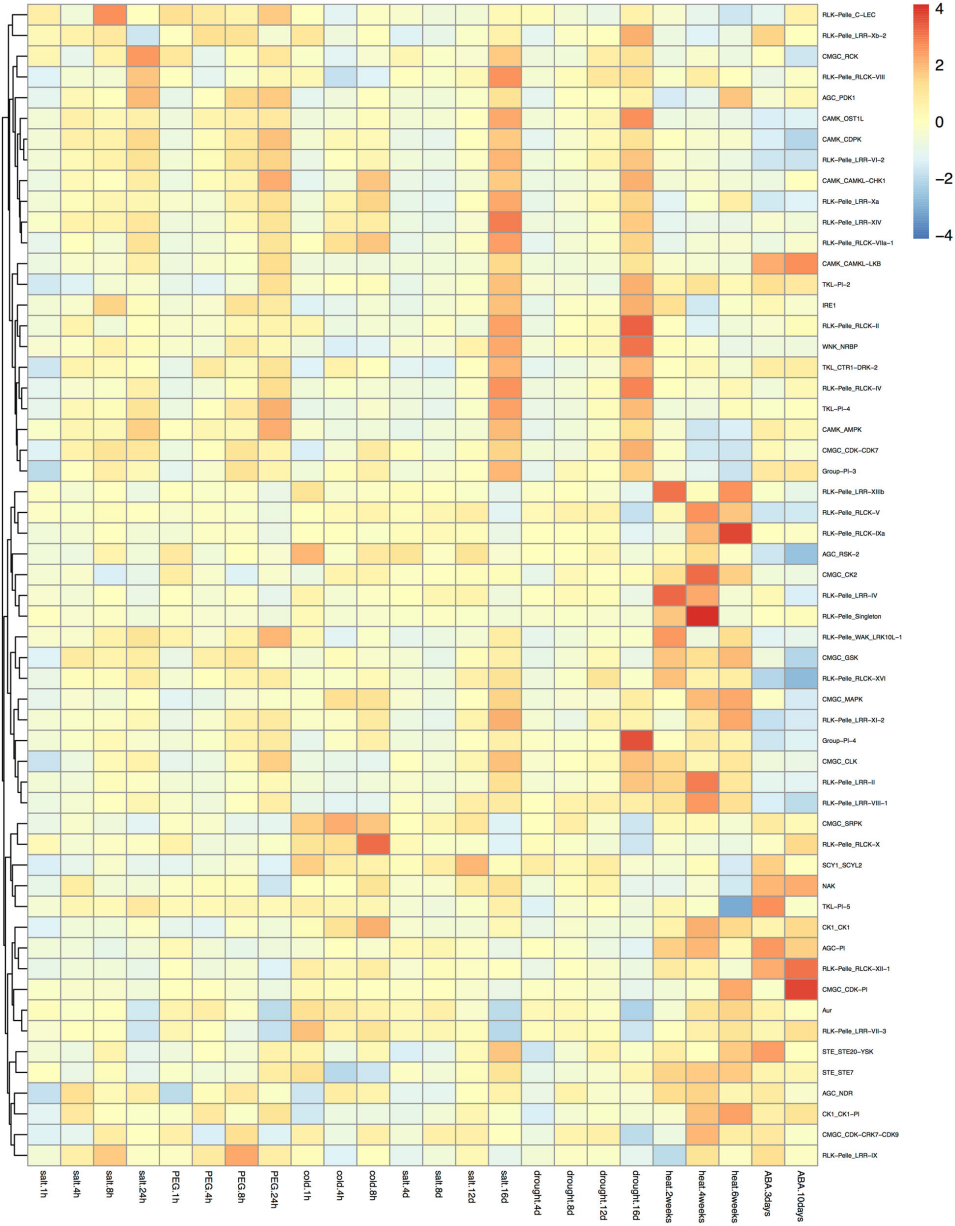
A heatmap of the expression data of 56 different grapevine kinase families in response to salt, PEG, cold, drought, heat, and ABA. The heatmap was generated using the R package pheatmap. The color scale represents expression levels, with red indicating high expression levels and blue indicating low levels

PKs play key roles in plant responses to various environmental stresses^3,24,37^. Therefore, the publicly available grapevine gene expression data from NCBI for responses to salt, PEG, cold, drought, heat, and ABA treatments (accessions GSE31594, GSE31677, GSE31675, and GSE31662) (Supplementary Table S8) were analyzed. Expression data covering 231 grapevine PK genes from 56 families indicated that half of the families exhibited significant up-regulation or downregulation in response to specific stresses (Fig. 5). Based on a cluster analysis, we found that some of these families were induced by multiple similar stress treatments. For example, AGC_PDK1 and RLK-Pelle_LRR-II were induced by salt, PEG, and heat. CAMK_CAMKL-CHK1, IRE, RLK-Pelle_LRR-VI-2, RLK-Pelle_RLCK-IV, and TKL-Pl-4 were upregulated in response to salt, PEG, and drought. Several PK families, such as Aur, RLK-Pelle_LRR-XIIIb, RLK-Pelle_LRR-IV, and RLK-Pelle_LRR-VII-3, were downregulated in response to salt, drought, and PEG stress. Other families exhibited contrasting expression patterns in response to different treatments. CMGC_CDK-Pl, RLK-Pelle_LRR-XIIIb, and RLK-Pelle_RLCK-IXa were downregulated in response to salt, PEG, and drought treatments but upregulated in response to heat stress.

**Fig. 6:**
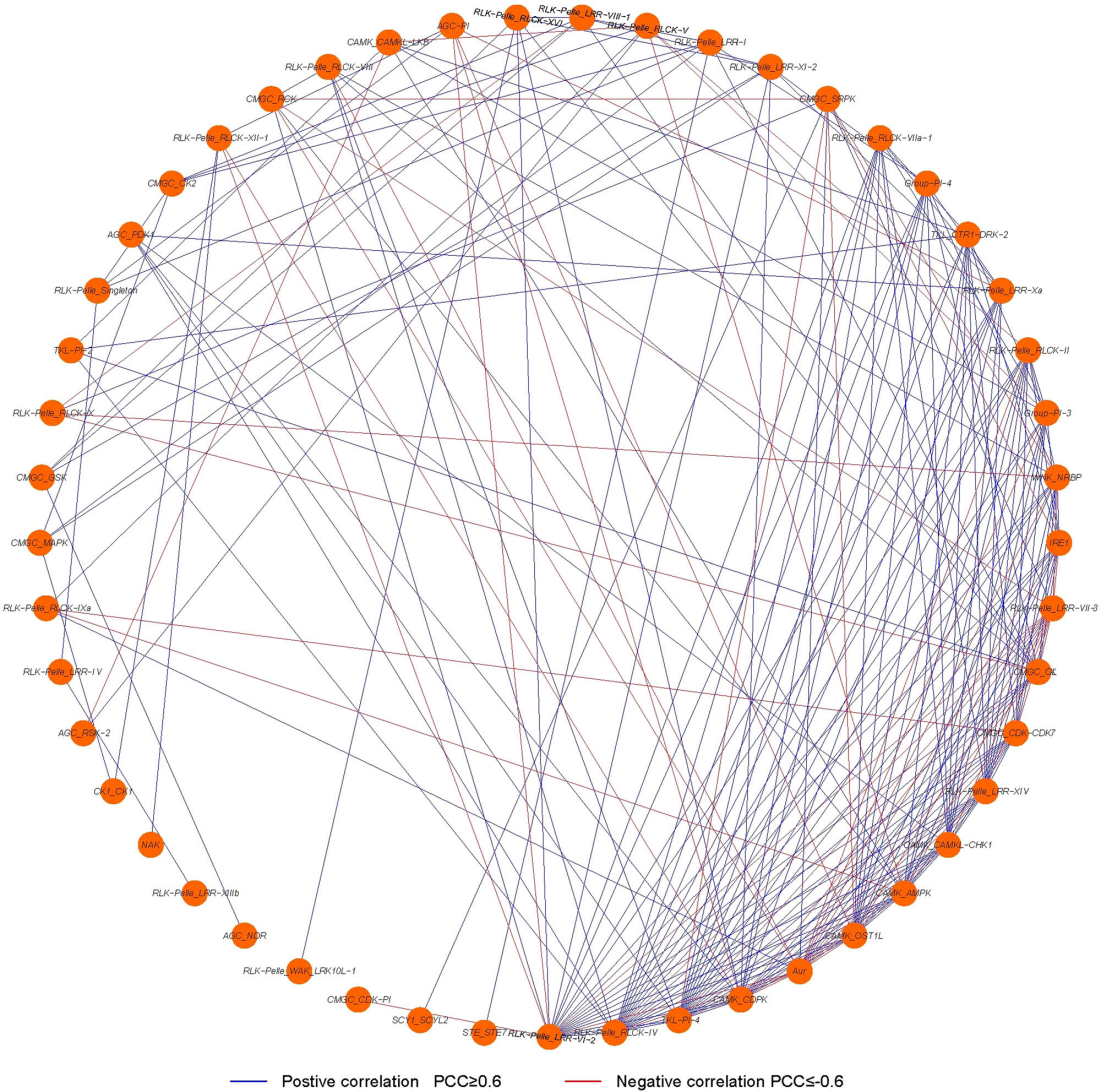
The coexpression network of grapevine PK families in response to salt, PEG, cold, drought, heat and ABA treatments. The nodes represent families, and the edges between nodes represent coexpression correlations of family pairs. Different edge line colors represent either positive (blue) or negative (red) correlations

A coexpression network was constructed in order to discover the mutual relationships between grapevine PK families by analyzing a large number of grapevine PKs with similar expression patterns under different conditions^41,42^. A coexpression network of grapevine families was constructed using the expression data described above to gain additional insight into the relationships and potential interactions between different grapevine PK families in response to six different treatments. The resulting coexpression network (Fig. 6) contained 49 nodes (grapevine PK families) and 222 edges (coexpression family pairs). Among 56 grapevine PK families, 49 were linked to each other through 222 edges, representing PCCs between coexpression events. Each node harbored a different number of regulatory edges, varying from 1 to 21. The TKL-Pl-4 family contained the maximum number of edges. Another five families, RLK-Pelle_RLCK-IV, WNK_NRBP, RLK-Pelle_LRR-VI-2, RLK-Pelle_LRR-VII-3, and RLK-Pelle_LRR-XIV, contained more than 10 edges, suggesting that these families may play central roles in the response of grapevine to different stress treatments. Among the 222 regulatory edges, 171 had significantly positive correlations, and the remaining 51 coexpression events had significantly negative correlations. A list of 20 kinase genes that were significantly induced (>two-fold, *P* < 0.05) by at least three different stresses (Supplementary Table S9) was constructed based on the expression and coexpression data. These 20 *PK* genes belonged to 4 different groups and 14 families. These PKs may play key roles in stress signaling pathways in grapevine and merit further investigation.

### RNA-Seq and qRT–PCR expression analysis of grapevine PK genes under drought and salt stresses

In addition to the public microarray data analysis, RNA-Seq analysis was also performed to analyze the functions of grapevine PKs under drought treatment. In total, 969 available grapevine PK genes showed their expression patterns in response to drought with FPKM values (Supplementary Table S10). We found that a large fraction (571/969) of the *PK* genes showed low FPKM values of less than 10 in all samples (Supplementary Fig. S4; Supplementary Table S10). Different *PK* genes showed various expression patterns. Some grapevine *PK* genes could be induced by drought treatment for at least one time point. However, some PK genes were downregulated under drought stress.

**Fig. 7:**
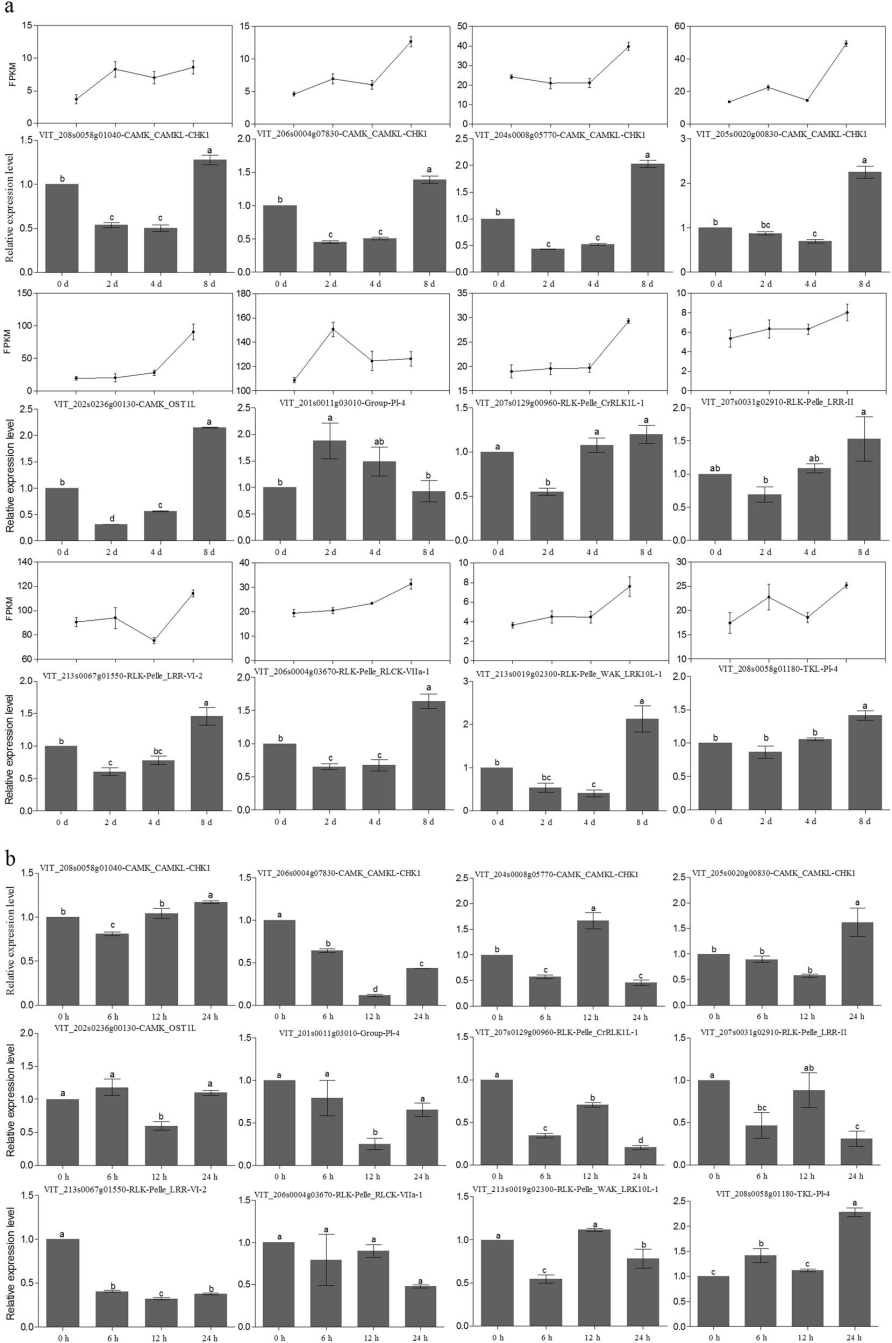
The expression patterns of grapevine PK genes under drought and salt stresses. **a** qRT–PCR expression data of 12 selected PK genes subjected to drought stress by withholding water for 0 (control), 2, 4, and 8 d after full water saturation. The RNA-Seq data of related grapevine PK genes are shown as FPKM values. **b** Quantification of expression levels of 12 grapevine PK genes in response to salt stress using qRT–PCR. Plants were subjected to salt stress by irrigating with 200 mM NaCl, and leaves were collected at 0 (control), 6, 12, and 24 h post salt treatment. The actin gene (*VIT_212s0178g00200*) was used as a reference gene to normalize gene expression levels. Values are the means ± SE of three replicates for each, and bars with different letters are significantly different at *P < *0.05 according to Duncan’s multiple range test

Twelve PK genes that were induced by drought in both the microarray (Supplementary Table S8) and RNA-Seq (Supplementary Table S10) analyses were selected for further confirmation using qRT–PCR after imposing drought stress (Fig. 7a). Specific primers for the 12 grapevine *PK* genes used in this study are listed in Supplementary Table S11. Interestingly, all these 12 PK genes were significantly induced by drought treatment at different time points. Most of them were highly upregulated at the 8-day time point only, with the exception of *VIT_201s0011g03010*, which was significantly induced only after 2 days of drought application. The expression patterns of most of the selected genes were similar in the RNA-Seq and qRT–PCR analyses, also indicating that the RNA-Seq data were reliable (Fig. 7a). We further analyzed the expression patterns of these 12 *PK* genes when plants were exposed to 200 mM NaCl (Fig. 7b). The 12 genes showed various patterns under salt stress. For instance, four kinase genes including *VIT_208s0058g01040*, *VIT_204s0008g05770*, *VIT_205s0020g00830*, and *VIT_208s0058g01180* showed higher expression levels than those of the control after exposure to NaCl for 12 or 24 h and were also upregulated under drought stress. Only one gene (*VIT_206s0004g03670*) had no significant changes under salt treatment. The remaining seven PK genes were downregulated at different time points. Overall, understanding the expression patterns of the grapevine PK genes might be valuable for improving the stress resistance of grapevine.

## Discussion

Plant kinases play a central role in signal transduction during plant growth and development and in plant responses to biotic and abiotic stresses. Comprehensive information on the grapevine kinome could provide further insight into the potential functions of PKs in grapevine.

### The grapevine genome possesses a large kinome

It has been reported that ~4% of the genes in a plant genome are kinase genes^43^. The grapevine genome contains 1168 putative grapevine kinase genes (Supplementary Table S1), accounting for ~3.7% of the total number of predicted genes. The number of grapevine PKs (1,168) is similar to the numbers in *Arabidopsis* (1008), maize (1241), and tomato (1436), but significantly more than the number previously reported (877) in grapevine^2,40,44^. It is, however, much smaller than the number of PKs in soybean (2,166)^3^ and larger than the kinomes in human^18^, mice^17^, oysters^45^, and the fungus *Wolfiporia cocos*^46^. The number of grapevine kinase families (121) (Fig. 1; Supplementary Table S3, Supplementary Fig. S1; Supplementary Fig. S2) is also similar to the numbers in *Arabidopsis* (119), rice (123), and soybean (122)^2,3^. The RLK-Pelle group is the largest kinase group in grapevine, accounting for 74.66% of the kinome, which is also similar to the proportion in other plant species^3,37,40^. Some grapevine PK families, however, had very few members. Members of these families may be involved in more basic cellular processes and less in processes associated with responses to stress^43^. Most grapevine PKs possessed only one kinase catalytic domain. Fifty-six of the grapevine PKs, however, contained two or three kinase domains (Supplementary Table S4). The grapevine kinases with two or three kinase domains may be required for specific substrates^3^. As reported for maize^40^, the intron number, pI, and MW of PKs within the same family were generally similar (Supplementary Table S3). Grapevine PK genes were unevenly distributed on all 19 chromosomes. However, many were tandemly arranged on a specific chromosome (Supplementary Table S3).

### Tandem and segmental duplications contributed greatly to the expansion of the grapevine kinome

In addition to whole-genome duplications, tandem and segmental duplication events have played major roles in the evolutionary expansion of gene families^19^. The existence of duplicated genes can also foster the acquisition of expanded functions for the new genes^47^. Tandem duplications tend to initiate changes in gene structure and function more quickly than other mechanisms of duplication^37^. Approximately one-third (395) of the grapevine kinome arose by tandem duplication, and 382 of the tandem duplication events occurred in members of the RLK-Pelle group of PKs (Fig. 2; Supplementary Table S5). The GO functional analysis revealed that the group of tandemly duplicated kinase genes as a whole was enriched in the GO categories “molecular function” and “biological process” (Fig. 3), suggesting that tandemly duplicated genes as a whole may play a central role in signaling pathways involved in plant development and responses to stress^3^.

Nearly two-thirds of the detected segmentally duplicated kinase genes (153/231) were also RLK-Pelle members. Additionally, based on the analysis of *Ka/Ks* ratios, it appears that the tandem and segmental duplication events in the grapevine kinome were subjected to purifying selection (Supplementary Table S6), which is also true for the soybean kinome^3^. The *Arabidopsis* kinome contains over 600 protein kinases that are RLK-Pelle group members, and the expansion of this group in *Arabidopsis* has also been largely attributed to tandem and segmental duplication events^14,48^. This type of expansion has also been found to be true for the kinomes of maize^40^ and soybean^3^. Several specific families, including RLK-Pelle_DLSV (158 members, the largest family in the grapevine kinome) and RLK-Pelle_LRK10L-2 (113 members), are responsible for the large size of the RLK-Pelle group in grapevine. The RLK-Pelle_DLSV family was also the largest family within the RLK-Pelle group of PKs in *Arabidopsis*, rice, and soybean^2,3^. The RLK-Pelle_LRK10L-2 family in grapevine, however, is much larger than that in many other plant species, including *Arabidopsis*, rice, maize, and soybean, but much smaller (206) than in *Eucalyptus grandis*^2^.

### The association of the grapevine kinome with plant development and plant stress response

Protein kinases have been reported to play critical regulatory roles in various biological processes in plants^11,49,50^. Previous studies have indicated that the grapevine CDPK gene family in the CAMK group and MAPK in the CMGC group are involved in a variety of developmental processes and plant responses to stresses^24,25^. Overexpression of a grape CDPK gene in *Arabidopsis* promotes plant growth and enhances ABA sensitivity during seed germination and early seedling growth^51^. Transgenic *Arabidopsis* overexpressing a CDPK gene (*VaCPK20*) from *Vitis amurensis* exhibited higher tolerance to heat, salt, cold, and drought, and a similar increase in stress tolerance was observed in transgenic grape cell cultures^52^. The expression patterns of different grapevine kinome family genes during development and in response to a variety of stresses suggest that the role of kinase families in these processes is diverse and may occur through different mechanisms. More than half of the grapevine kinase genes in the RLK-Pelle group were downregulated in most tissues during development, similar to the situation reported in maize organs^40^, suggesting that they may have negative regulatory functions. Many non-RLK groups, including CAMK, CK1, CMGC, STE, and TKL, however, exhibited high expression levels in different tissue samples during development (Fig. 4), suggesting that these kinase genes may play positive regulatory roles in plant growth, as reported in soybean^3^.

Approximately one-third of the rice *RLK-Pelle* *PK* genes expressed in the embryo and endosperm were reported to be regulated by abiotic stress, with the majority of them being upregulated by salt and drought^53^. Overexpression of *OsSIK1*, a putative *RLK* gene from rice, improved drought and salt stress tolerance in rice^54^. Several *Arabidopsis* *RLK-Pelle* genes were upregulated in response to osmotic, wounding, and UV-B stresses^37,55^. Overexpression of an *Arabidopsis* receptor-like protein kinase 1 (RPK1) in *Arabidopsis* regulated reactive oxygen species (ROS) homeostasis and increased drought tolerance, while a mutant, *rpk1-1*, lacking RPK1 function exhibited increased water loss^56^. Our analysis of the *Arabidopsis* kinome families revealed different expression patterns in response to salt and drought and that a portion of the families could be significantly induced by these stresses (Supplementary Fig. S5). Among the 56 grapevine kinase families that exhibited responses to a variety of stresses (Fig. 5), 23 belong to the RLK-Pelle group. Eighteen of the 23 families exhibited either up or downregulation in response to different stress treatments, indicating that the RLK-Pelle group may play important roles in plant responses to stresses. Interestingly, analysis of the different expression patterns under multiple treatments showed that about half of the 56 kinase families were induced by salt, and drought, which indicated that grapevine kinase genes might have important functions in responses to salt and drought stresses (Fig. 5). Based on the regulation of grapevine kinase genes by different stresses, a coexpression network of families was constructed to elucidate the relationships between different families of PK genes (Fig. 6). A kinase gene coexpression network for several kinase families in soybean^3^ suggested that expression of those families was associated with either broad or specific stress signal transduction pathways. In our study, several grapevine RLK-Pelle families were highly coexpressed, which indicates that they may interact in plant stress response signaling. Based on the expression data of 231 grapevine PK genes under different treatments (Supplementary Table S8), the genes that were significantly induced (>two-fold, *P* < 0.05) by multiple treatments would be strong candidates for further investigation.

Gene expression data can provide only indirect evidence of gene function. Thus, due to the large size of the kinase gene family in plants and the varying levels of functional redundancy within this family, confirming the functions of individual genes is a daunting challenge. Family-level analyses of expression may help to quickly identify candidate targets that are deserving of more detailed study. Since only a few grapevine kinase genes have been functionally characterized, characterization of the entire grapevine kinome, its gene expression patterns during grapevine development and in response to stress treatments, and the coexpression network of families in response to a variety of stress treatments provide fundamental information for the selection of candidate genes for further study. The 20 selected kinase genes, which were significantly induced by multiple treatments, might play key roles in stress signal transduction (Supplementary Table S9). Among the 20 genes, 7 belonged to the CAMK group and 11 were RLK-Pelle group members. These results suggest that these two groups may be involved in plant responses to a variety of stresses^25,37,53^. Five of the 20 genes are members of the CAMK_CAMKL-CHK1 family, which are also known as CBL-interacting protein kinases (CIPKs) and have been demonstrated to play an essential role in plant response to a variety of stresses in other plants^11,57,58^. Our RNA-Seq and qRT–PCR analysis verified four PK genes (*VIT_208s0058g01040*, *VIT_204s0008g05770*, *VIT_205s0020g00830*, and *VIT_208s0058g01180*) induced by both drought and salt stresses, and three of them were CAMK_CAMKL-CHK1 members, suggesting that these genes might play key functions in response to environmental stresses and deserve further functional investigation (Supplementary Table S10; Fig. 7). Our information will greatly assist in developing a comprehensive understanding of this very large gene family. In addition, this study will help to clarify the roles of specific members in growth and development as well as environmental stress response and adaptation (tolerance) in grapevine.

## Electronic supplementary material

Supplementary Figures(DOCX 2342 kb)

Supplementary Table S1(XLSX 181 kb)

Supplementary Table S2(XLSX 73 kb)

Supplementary Table S3(XLSX 318 kb)

Supplementary Table S4(XLSX 55 kb)

Supplementary Table S5(XLSX 72 kb)

Supplementary Table S6(XLSX 147 kb)

Supplementary Table S7(XLSX 948 kb)

Supplementary Table S8(XLSX 148 kb)

Supplementary Table S9(XLSX 53 kb)

Supplementary Table S10(XLSX 119 kb)

Supplementary Table S11(XLSX 57 kb)
