# Supplementary material for: The grapevine kinome: annotation, classification and expression patterns in developmental processes and stress responses
Source: Hortic Res. 2018 Apr 1;5:19. doi: 10.1038/s41438-018-0027-0 (PMC5878832; doi:10.1038/s41438-018-0027-0)
Supplement: Supplementary file 1 — Supplementary Figures(DOCX 2342 kb) [file 41438_2018_27_MOESM1_ESM.docx]

**The grapevine kinome: annotation, classification and expression patterns in developmental process and in response to stresses**

**Running title: The grapevine kinome**

Kaikai Zhu^1,2^, Xiaolong Wang^1,2^, Jinyi Liu^1^, Jun Tang^3^, Qunkang Cheng^4^, Jin-Gui Chen^5^ and Zong-Ming (Max) Cheng^1,2,^*

^1^ College of Horticulture, Nanjing Agricultural University, Nanjing, Jiangsu 210095, China

^2^ Department of Plant Sciences, University of Tennessee, Knoxville, TN 37996, USA

^3^ Jiangsu Key Laboratory for Horticultural Crop Genetic Improvement, Institute of Horticulture, Jiangsu Academy of Agricultural Sciences, Nanjing, Jiangsu 210014, China

^4^ Department of Entomology and Plant Pathology, University of Tennessee, Knoxville, TN 37996, USA

^5^ Biosciences Division, Oak Ridge National Laboratory, Oak Ridge, TN 37831, USA

* Correspondence: zmc@njau.edu.cn, zcheng@utk.edu

**Supplementary data**

Supplementary data are available online.

Supplementary Fig. S1. Classification and phylogenetic analysis of grapevine protein kinases.

Supplementary Fig. S2. Classification and phylogenetic analysis of grapevine protein kinases by families.

Supplementary Fig. S3. Comparison of the frequency distribution of relative *Ka/Ks* ratios between segmental and tandem duplication events.

Supplementary Fig. S4. RNA-Seq analysis of grapevine PK genes under drought stress for 0, 2, 4 and 8 d.

Supplementary Fig. S5. A heatmap of the expression data of 119 different *Arabidopsis* kinase families in response to salt and drought.

Supplementary Table S1. Kinase domain annotation of typical grapevine protein kinases.

Supplementary Table S2. Kinase domain annotation of atypical grapevine protein kinases.

Supplementary Table S3. Family classification of grapevine protein kinases and their related information.

Supplementary Table S4. List of 56 grapevine protein kinases containing multiple kinase domains.

Supplementary Table S5. List of 395 tandemly duplicated grapevine protein kinases.

Supplementary Table S6. Segmental and tandem duplication events and *Ka*/*Ks* values of grapevine protein kinases.

Supplementary Table S7. Normalized gene expression values of 1,109 grapevine PK genes in 54 different tissues.

Supplementary Table S8. Normalized gene expression values of 231 grapevine PK genes in response to different stress treatments.

Supplementary Table S9. Kinase genes in the grapevine kinome that are significantly induced by at least three abiotic stresses.

Supplementary Table S10. Average FPKM expression values of 969 grapevine kinase genes under drought stress.

Supplementary Table S11. List of primer Sequences used for qRT-PCR analysis in this study.

**Fig.S1**

**Supplementary Fig. S1.** Classification and phylogenetic analysis of grapevine protein kinases. The phylogenetic tree was constructed by MEGA6 with the Neighbor-joining (NJ) method using kinase domain sequences. Bootstrap values were calculated using 1,000 replicates. Families are highlighted with various colors.

**Fig. S2**

**Supplementary Fig. S2.** Classification and phylogenetic analysis of grapevine protein kinases by families. The phylogenetic tree was constructed using MEGA6 with the Neighbor Joining method based on the kinase domain sequence. Bootstrap values were calculated by 1,000 replicates. Families are highlighted with various colors.

**Fig.S3**

**
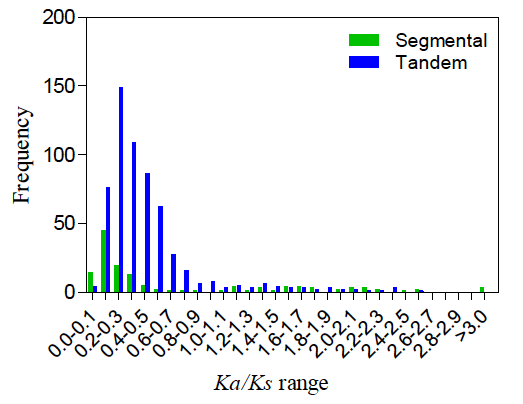
**

**Supplementary Fig. S3.** Comparison of the frequency distribution of relative *Ka/Ks* ratios between segmental and tandem duplication events. The X-axis denoted average *Ka/Ks* per unit of 0.1 and Y-axis denoted frequency.

**Fig.S4**

**Supplementary Fig. S4.** RNA-Seq analysis of grapevine PK genes under drought stress for 0, 2, 4 and 8 d. Log_2_ (FPKM+1) values were performed according to the color scale. The Heatmap was constructed using R package pheatmap.

**Fig S5**


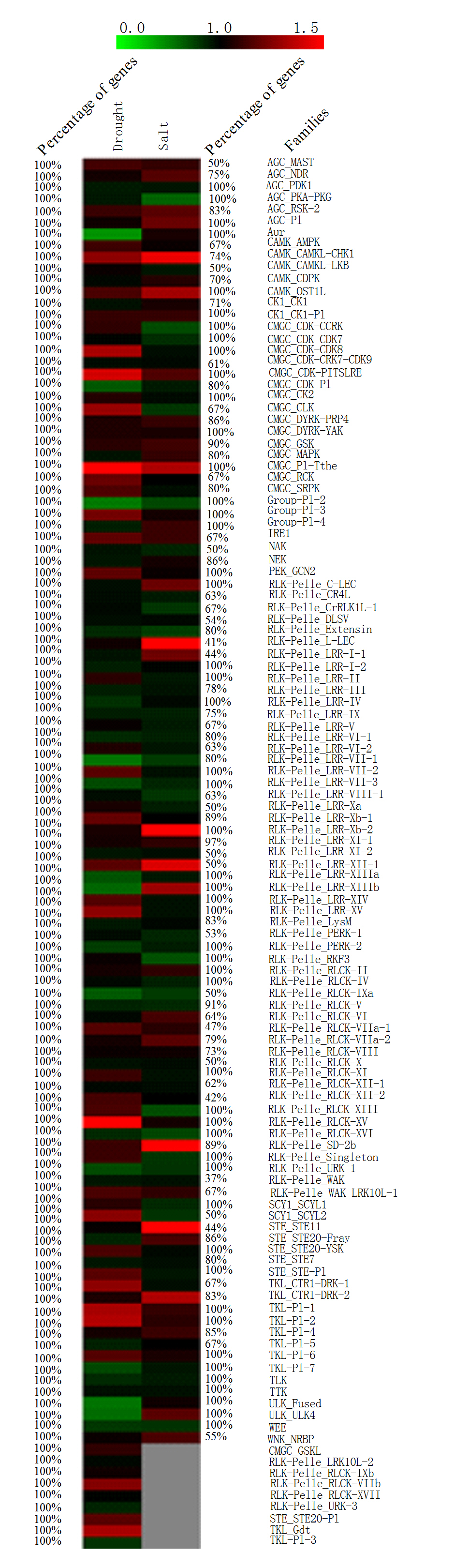


**Supplementary Fig. S5.** A heatmap of the expression data of 119 different *Arabidopsis* kinase families in response to salt and drought. The heatmap was generated using MeV. The color scale represents expression levels, red indicating high expression levels and green indicating low levels.
